# Empirical study of the relationship between design patterns and code smells

**DOI:** 10.1371/journal.pone.0231731

**Published:** 2020-04-16

**Authors:** Mahmoud Alfadel, Khalid Aljasser, Mohammad Alshayeb

**Affiliations:** 1 Department of Computer Science and Software Engineering, Concordia University, Montreal, Canada; 2 Information and Computer Science Department, King Fahd University of Petroleum and Minerals, Dhahran, Saudi Arabia; University of Pisa, ITALY

## Abstract

Software systems are often developed in such a way that good practices in the object-oriented paradigm are not met, causing the occurrence of specific disharmonies which are sometimes called code smells. Design patterns catalogue best practices for developing object-oriented software systems. Although code smells and design patterns are widely divergent, there might be a co-occurrence relation between them. The objective of this paper is to empirically evaluate if the presence of design patterns is related to the presence of code smells at different granularity levels. We performed an empirical study using 20 design patterns and 13 code smells in ten small-size to medium-size, open source Java-based systems. We applied statistical analysis and association rules. Results confirm that classes participating in design patterns have less smell-proneness and smell frequency than classes not participating in design patterns. We also noticed that every design pattern category act in the same way in terms of smell-proneness in the subject systems. However, we observed, based on the association rules learning and the proposed validation technique, that some patterns may be associated with certain smells in some cases. For instance, Command patterns can co-occur with God Class, Blob and External Duplication smell.

## 1. Introduction

Design patterns (DP), are recurring solutions to common software design problems. DPs aim to improve reusability and reduce coupling [1]. DPs can ease communication among team members by using terminology instead of using traditional explanations [2]. Gamma et al. [3] identified 23 design patterns which can be divided into three categories: creational, structural and behavioral. Creational design patterns are those that deal with creating objects so that the created objects serve a purpose that is suitable to the situation. Structural design patterns are design techniques that facilitate software design by identifying simple ways to realize relationships among the different entities. Behavioral design patterns are communication patterns. Design patterns or design motifs refer to several classes of different roles. Each design motif plays role in source code [4]. DPs have an impact on software quality attributes such as maintainability, reusability and fault-proneness [3, 5]. Design patterns may affect software quality attributes positively or negatively [1, 2]. Design patterns may degrade the system performance and make code unnecessarily complex when they are not used in a proper manner [1]. Different studies reported different results for the impact of design patterns on software quality. Vokáč [6] found that participant design pattern classes have fewer defects. Additionally, Prechelt et al. [1] reported that design patterns can positively affect maintenance aspects. On the other hand, other studies [7–10] reported negative impact of design pattern on software quality. Therefore, the question about design patterns impact on software quality is still open due to such inconclusiveness.

Code smells are problems that appear in a fragment of code that make software hard to maintain and change [11]. In 1999, Fowler defined code smells as signs of design problems that can hamper software maintenance [11]. However, code smells, unlike design pattern, are not patterns but they are signs where developers can look for a concrete issue. Therefore, code smells can suggest places in code required to be examined more thoroughly. Similar to design patterns, researchers reported different results about the impact of code smells on various quality aspects. Li and Shatnawi [12] found that code smells and defect density are correlated. However, studies in [13–15] have shown that smells are useful indicators to explain maintenance issues. Several factors such as software domain and experience of programmer have been used to detect code smells [16, 17]. Hence, it is important for researchers to come up with other mechanisms to detect bad smells. Therefore, expanding other factors could help practitioners in better understanding bad smells relation with code.

Some recent works studied design patterns and code smells in different ways. The majority of them discussed the topic from refactoring points. For instance, studies [18–20] proposed tools that suggest fragments need refactoring. Other studies [21, 22] investigated the relation between design patterns and code smells in terms of their structural relationship. Furthermore, Wendorff [9] reported cases where design patterns may have negative impact on attributes like maintainability which may lead to introduce bad smells. McNatt and Bieman [23] reported that some design patterns such as Command, Proxy, Bridge and Observer may degrade the system performance and make code uncontrolled when they are not used in a proper manner.

Design patterns are associated with good software design and code while bad smells indicate a lack of design or code flaws. DPs and bad smells represent antagonistic structures; therefore, they are rarely investigated in the same research context. Considering the conflicting results of the impact of DPs on quality attributes and having ambiguous relations that relate code smells with maintainability aspects due to design patterns applications; the connection between DPs and code smells with special focus on their co-occurrences deserves more investigation. Moreover, only a few studies have investigated the direct relationship between DPs and code smells [24]. Thus, more studies are needed to rigorously investigate and contrast a generalized evidence of the impact of design patterns on code smells. Also, we explore the potential relationship between code smells and DPs at different granularity levels.

Descriptions of some patterns could reveal potential associations with smells. For example, the Strategy pattern creates a set of objects in classes that represent algorithms. However, data which might be needed by the objects is decoupled. Hence, such situation could introduce Feature Envy smell in the classes that hold data. The Factory Method pattern has a similar situation of the Strategy pattern. When a method in Factory Method pattern creates objects from other classes, it sends a set of parameters to set up an instance. Hence, the pattern could result in an association with Long Parameter Lists smell and Blob smell. Such hypotheses motivated us more to conduct this study to explore if there is a potential relationship between design patterns and code smells. In this study, we initiate an assessment of the relationship between both concepts using Java language.

The study of the relationship between design patterns and code smells can uncover other factors that could help code reviewers to have better understanding of code quality. The analysis of the relation between design patterns and code smells can provide software designers with the knowledge and guidance to employ design patterns properly. Understanding the relationship can provide code reviewers with indications about the occurrence of smells over the code. For example, knowing patterns that are not mutually connected with smells could help reviewers and testers to focus their efforts on other parts of the code. On the other hand, potential co-occurrences of design patterns and bad smells could also help testers focus on parts that might have smelly design pattern classes. Studying the relation between DPs and code smells can help in understanding their associations with other parts of the code structure. Inspired by the study of Walter and Alkhaeir [24], we focus on the occurrence of code bad smells in code fragments that are part of design patterns, where more systems, design patterns, and code smells are analyzed. Also, we consider smell frequency factor in studying the potential co-occurrence. Moreover, we test the relationship between DPs and smells from the category level perspective. Lastly, we manually validate the significant cases that show potential relationships in order to gain more insights and draw more generalizable evidence about the code smells occurrence in DPs. The work presented in this paper has been extracted from a master thesis [25].

This study has four main contributions: 1) a confirmation study of previous works on the differences between classes involved in design patterns and classes that are not involved in design patterns in terms of smell proneness and smell frequency at class level, 2) an empirical evaluation of the differences in smell-proneness among classes that involve design patterns at DP category level, 3) an empirical evaluation of the differences in smell occurrences among classes that participate in a design pattern at motif level and 4) provide the dataset used in this study publicly for future replication.

The remainder of this paper is structured as follows. The related work is presented in Section 2. Section 3 describes the experiment setup. Section 4 highlights the experiment results while a discussion of the results is presented in Section 5 along with the limitations of the proposed work. Finally, Section 6 concludes the paper and recommends future research directions.

## 2. Related work

In this section, we review the related work with a special focus on the impact of DPs on software quality attributes, the impact of code smells on software quality attributes and the relationship between DPs and code smells.

### 2.1 The impact of design patterns on software quality attributes

Researchers investigated the impact of DPs on several quality attributes such as maintainability, change-proneness, performance and fault-proneness. Prechelt et al. [1] performed an experiment to investigate software maintenance that uses several design patterns and compared them with other scenarios with simpler alternatives. They found that, in many cases, design solutions in which GoF’s patterns are employed are easier to maintain than their corresponding simple solutions. Nonetheless, the authors also found that there are situations in which the use of design patterns made the maintenance of the program harder. They also confirmed that compared to a straightforward solution, design patterns may require additional maintenance tasks. Vokac et al. [26] replicated Prechelt et al.’s [1] study, finding that the Visitor pattern led to a high cost in terms of development time and poor accuracy. They also found that Decorator eases maintenance, despite the fact that it is hard to trace the control flow of the program and hence increases the effort required to understand it. Therefore, although its maintainability is good, it decreases understandability. Several studies [27–29] have been conducted to replicate the study in [1]. Some studies [27–29] found that DPs have a negative impact on maintainability while others (e.g. [30]) found no clear trend of any impact and recommended a deeper and a practical analysis be conducted. In addition, Garzas et al. [31] found that pattern-based designs make understandability and modifiability harder. On the other hand, Hegedus et al. [32] evaluated the impact of some DPs in JHotDraw systems on maintainability and found that DPs can affect maintainability positively and lead to improvements in the code.

Lutz Prechelt et al. [33] conducted a controlled experiment to investigate whether maintainer can perform better and fast if DPs in program code are explicitly documented using comments as compared when having only well-documented program without reference to DPs. They stated that patterns-based maintenance tasks were faster or with fewer problems as compared to the other way. Aversano et al. [34] performed a study to evaluate the relationship between DPs and change-proneness. The results show that DPs are more prone to changes under the condition that DPs play a significant role in system functionality. On the other hand, Gatrell et al. [35] found that some DPs participant classes are more prone to changes than non-participant classes. Bieman et al. [36] investigated the relationships between software change-proneness and design structure. Five open-source systems were used to conduct the experiment. Design patterns, size of a class and class inheritance participation were used to recognize design structure. The results of the study show that classes which participate in design patterns are more change-prone than other classes. This result may be justified by the fact that design patterns provide essential key responsibilities and functionality to the system. This may explain why classes that participate in design patterns are more change prone than other classes, relatively. Change-proneness in classes which participate in design patterns may be an indication of the co-occurrence of design patterns with either Divergent Change or Shotgun Surgery code smells, as defined by Fowler et al. [11]. Aversano et al. [34] concluded that design patterns appear to produce code which has a greater resistance to change. Moreover, they discovered that patterns are more suitable for applications which usually change more often.

The literature refers to some DPs which contribute to the presence of faults. Several studies discuss this issue [6, 37–39]. Vocak [6] studied the relationship between DPs and the number of faults using C++ systems. The results show different trends for different patterns. For example, Singleton and Observer were found to have more faults, due to their huge structural associations. In contrast, Factory Methods were subject to fewer faults because Factory Method classes are loosely coupled to the system structure. Additionally, no clear trend was found for the Template Method and Decorator patterns. The relationship between DPs and fault-proneness was investigated by Gatrell and Counsell [37]. The authors observed that classes participating in DPs are more prone to faults than classes not participating in DPs, especially for the Adaptor, Singleton and Template Method. Their study was conducted on ten Gang of Four (GoF) patterns using C# systems. Ampatzoglou et al. [38] evaluated the relationship between eleven DPs and fault frequency using Java systems. The results show both positive and negative relationship of the analyzed patterns to defect frequency. For example, the Adaptor pattern correlates positively with the Defect frequency, whereas Observer is negatively correlated with the Defect frequency.

From the previously mentioned studies, it can be observed that different studies reported different results for the impact of design patterns on software quality and the question about design patterns impact on software quality is still open due to such inconclusiveness.

### 2.2 The impact of code smells on software quality attributes

Code smells are perceived to lead to maintenance difficulties in software systems [13, 14, 40–42]. In addition, some studies claim that classes which have code smells are liable to be more change prone [40, 43, 44] and have more defects [40, 45–48] than classes that do not have code smells.

Olbrich et al. [40] studied the impact of God and Brain class smells on change size, change frequency and defects and found that classes with these code smells have more change size, change frequency and defects. The results of this study were further validated with more smells in [49, 50]. The studies [12, 43, 51] are in line with the findings of [40]. Interestingly, Olbrich et al. [40] found that when their results were normalized with respect to size i.e. Line of Code (LOC), the results no longer hold under the assumption that classes which are involved in code smells i.e. God Class and Brain Class have a ratio of functionality similar to other classes, on average. Thus, they concluded that code smells are not generally harmful and that code smells in classes may be an efficient way to organize code, providing that these smelly classes are constructed intentionally.

Abbes et al. [42] conducted an empirical study on the impact of anti-patterns i.e. Blob and Spaghetti Code, on program understandability. Their conclusion outlined that only one occurrence of Blob or one occurrence of Spaghetti does not significantly decrease program understandability. On the other hand, a combination of both Blob and Spaghetti affects program understandability and thus affects maintainability.

Jafaar et al. [45] studied the impact of design pattern classes that have dependencies with non-design pattern classes on defects and change-proneness. This study was conducted in the case of anti-pattern classes and non-anti-pattern classes. The results of their work show that classes which have dependencies on anti-patterns produce more defects those which have dependencies on design patterns. However, the results in [45] vary from one anti-pattern to another depending on the smell in addition to the analyzed software subjects. Khomh et al. [44] conducted an experimental study concerning the impact of anti-patterns on change-proneness and fault-proneness. They concluded that classes with anti-patterns are more change-prone and fault-prone. In addition, they found that structural changes affect classes participating in anti-patterns more than other classes.

Research community dedicated good efforts for understanding the code smells taking place in production code [52]. For instance, Tufano et al. [52] conducted an empirical study on a large set of open source projects (i.e. 200 projects). The objective of their study is to understand when and why code starts to smell. Therefore, the study was conducted over change history. Their results contradict common previous studies, where most of smells in the projects are initiated when the artifacts are created and not when they evolve. In addition, 80% of smells remain in the projects and only 9% of removed instances happened for refactoring purposes.

Code smells are not always bad; it depends on the situation in which code smells occurred in the source code. Hence, considering more smells in code individually could lead to different insights. Therefore, it would be interesting in the context of this study to analyze individual code smells code. The literature shows that studies investigated code smells and their relations with change-proneness, defects and understandability of systems.

### 2.3 The relationship between design patterns and code smells

The work that is most related to our study is the study of Walter and Alkhaeir [24]. In their work, they conducted an empirical study to evaluate the relationship between DPs and code smells at the class level. The authors examined this relation using 10 GoF’s design patterns and 7 code smells. They used two open-source projects, JfreeChart and Maven, with many subsequent releases of each project. The results indicate that the presence of DPs is not strongly associated with code smells, i.e., classes that participate in design patterns most likely are not smelly classes. However, these observations are more supported for certain patterns, such as Singleton and less supported for others, such as Composite. No clear trend was found regarding the evolution of smells over the analyzed pattern-based systems. The data provided by the study is limited to the class level and does not cover the category and role level of DPs. This is due to the limitation of the design patterns detection tool used in their study [53]. Generally, their findings designate that design pattern classes are connected to lower number of smells. Inspired by their study and supported by the fact that different design patterns may be associated with different code smells, in this study, we consider more systems, design patterns, and code smells. Furthermore, we consider smell frequency factor in studying the potential co-occurrence between design patterns and code smells. Moreover, we test the relationship between DPs and smells from the category level perspective. Lastly, we manually validate the significant cases that show potential relationships in order to gain more insights and to identify if a specific code smells or group of code smells is associated with a specific design pattern or group of design patterns. Unlike Walter and Alkhaeir study, we used a public manually validated design patterns dataset in order to increase the reliability of the results. Although our overall results show close findings to their study [24], we observed that some patterns are associated with certain smells. For instance, The Command pattern is found to be associated to the Blob, External Duplication and God Class smells. We conclude that there is a relationship between the existence of specific design patterns and bad smells in specific scenarios.

Codabux et al. [54] conducted an empirical study on the relation between code smells with class-level (micro pattern) and method-level (nano-pattern) traceable code patterns. They found that Immutable and Sink micro patterns are more frequent in classes having code smells. Cardoso and Figueiredo [55] performed an exploratory study to identify instances of co-occurrences of design patterns and bad smells using five systems. They found that the co-occurrences of Command with God Class and Template Method with Duplicated Code. Sousa et al. [56] conducted a case study with five Java systems to investigate the cooccurrence of design patterns and bad smells using software metrics. They focused on investigating if the use of design pattern reduces bad smell occurrence. They found that the application of design pattern not necessarily avoid bad smell occurrences. Sousa et al. [57] conducted a systematic literature mapping study on the relationship between design patterns and bad smells. They identified 16 primary studies and classified them into three approaches 1) impact on software quality, 2) refactoring 3) co-occurrence.

Khomh [21] proposed a quality model that takes into consideration several styles of design, including design patterns, code smells and anti-patterns. The author stated that the strength of the design structure is important in measuring the quality characteristics. Therefore, he analyzed how DPs and code smells can impact the quality characteristics, fault- and change- proneness. The results show that the model has a better and more accurate evaluation over the traditional metrics-based model.

Some works studied the refactoring techniques and tools that target improving software quality [58, 59]. Seng et al. [58] discussed the importance of maintenance that helps to eliminate code smells. Thus, they proposed an automa tic search-based approach to suggest the possible refactoring segments based on design patterns in the code of systems. The approach tends to improve the structure of software design without changing its behaviors. The approach was validated using only one open-source system called JhotDraw. Alshayeb [59] investigated the impact of software quality on refactoring to design patterns and found that there are no clear trends about refactoring or refactoring to patterns in quality improvements.

In addition to confirming the findings of prior studies, this study is different from existing studies in different ways. In particular, unlike the work of Walter and Alkhaeir [24], we address both smell-proneness and smell frequency whilst their work only considers smell-proneness. Also, we analyzed 20 design patterns and 13 code smells whilst their work examined 9 design patterns and 7 code smells. Furthermore, our work also targets different design pattern levels: class level, category level, motif level and role level (source-code based technique). Furthermore, for validation purposes, we use 10 different Java open source systems with the validated design pattern data. Lastly, we manually using a validation technique confirm a case of design patterns and code smells co-occurrence.

## 3. Empirical study setup

The main objective of this study is to explore if there is a relationship between design patterns and code smells. We define the following sub-objectives:

1. To empirically evaluate code smell-proneness and smell frequency in design pattern classes versus non-design pattern classes at the class level.
2. To empirically evaluate code smell-proneness in design pattern classes versus non-design pattern classes in design patterns categories (creational, structural and behavioral).
3. To empirically evaluate code smell-proneness for individual design motifs. This leads to identify roles of significant design patterns, if any.

The next sections present the details of the empirical study including: the research questions, the analyzed design patterns and code smells, data collection, research methodology and measurement tests.

### 3.1 Research questions

To achieve the objective of this study, we evaluate the impact of design patterns on code smells at the following different granularity levels:

- **Class Level**: this level empirically evaluates the differences in terms of smell-proneness and smell frequency between classes involved in design patterns and classes that are not involved in design patterns i.e. (Smelly Design Pattern Classes (SDP) versus Smelly Non-Design Pattern Classes (SnDP)).
- **Category Level**: this level empirically evaluates the differences in smell-proneness among classes that involve different categories of design patterns.
- **Motif Level**: this level empirically evaluates the differences in number of smells occur among classes that involve a specific single design motif individually. In addition, in this level we identify the roles of significant design motifs, if any.

In relation to the aforementioned levels, we formulate the following research questions:

RQ1: Are design pattern classes more smell-prone and more frequent than non-design pattern classes?

RQ2: Do code smells have significant differences in terms of proneness when they are present in the different categories of design pattern classes?

RQ3: Are the participant classes in a specific individual design motif more smell-prone for a specific smell?

### 3.2 Research methodology

**Fig 1:**
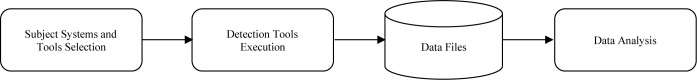
Research methodology.

To achieve the research objective, we follow the methodology detailed in Fig 1. The methodology phases are: (1) the selection of subject systems and detection tools (2) the execution of the tools to obtain the related results of design patterns and code smell data and (3) data analysis and data mining.

In the first phase, we selected the systems to be used for analysis based on the P-Mart repository [60]. In addition, we selected the design patterns and code smell tools. The inFusion tool [61] was selected for code smell detection while the P-Mart repository was used for design pattern data. We kept the default values of the configuration parameters for code smell detection tool for possible replication.

In the second phase, we collected code smells and design pattern data of each system. For the design patterns, we used the available XML files in the P-Mart repository for each system and parsed them to get each design pattern class along with its corresponding design patterns. Hence, 10 files are resulted in this step. For code smells, we ran the tool over each system and collected the smelly classes along with the number of smells in each class with the type of smell. This step also resulted in 10 files for each system. Next, for each system, we matched both files of design patterns and code smells to be compared later. Therefore, we created one file per project to store the output results of the design patterns and their corresponding code smells. This makes the needed information of design patterns and code smells available for each class in each file in the systems. Each file has 25 columns; the first column is the class name followed by the occurrence of the corresponding design pattern in that class, if any. Column number three indicates the code smell occurrence (0 or 1) followed by the column showing the number of smells in that class. The fifth column specifies the type of smells the class may have. The remaining 20 columns refer to the corresponding design patterns types classified based on the categories of design patterns (i.e., Creational, Structural, and Behavior).

In the last phase, we performed statistical data analysis and data mining using the data in the stored files to analyze the results. Section 3.3 and Section 3.4 discuss more details of the data collection and statistical tests used in this study. Next, we explain the data collected in this study.

### 3.3 The used design patterns and code smells

In this work, we used 20 design patterns out of the 23 proposed by Gamma et al. that are available in the P-Mart repository [60]. Table 1 lists the design patterns used in this study.

**Table 1:** The used design patterns.

Name	Category
Abstract Factory, Builder, Factory Method, Prototype, Singleton	Creational
Adapter, Bridge, Composite, Facade, Decorator, Proxy	Structural
Command, Iterator, Mediator, Memento, Observer, State, Strategy, Template Method, Visitor	Behavioral

Several classifications have been associated with bad smells such as code smells and design smells [62]. Antipattern is another concept which is associated to code smells. Jaafar et al. [63] stated that code smells are more related to the inner scope of classes while anti-patterns are related to the relationships among classes. In this work, the used code smell detection tool was able to find 13 code smell types in the used dataset. Therefore, we perform our analysis on those thirteen code smells: seven smells that are defined by Fowler [11] and six smells that are defined by Lanza and Marinescu [64]. Table 2 lists these smells with their abbreviations.

**Table 2:** The used code smells.

Name	Acronym
Data Class	DC
Data Clumps	DCl
Refused Parent Bequest Class	RPB
Schizophrenic Class	SC
Blob Methods	BL
Intensive Coupling	IC
Sibling Duplication	SD
Internal Duplication	ID
External Duplication	ED
God Class	GC
Feature Envy	FE
Tradition Breaker	TB
Message Chains	MC

### 3.4 Data collection

This section describes the data used in this research along with the collection process.

#### 3.4.1 Research data

To conduct the empirical experiments, we require data that satisfies the following: 1) classes which are participating in patterns, (2) classes which are participating in code smells. In addition, the data should:

1. be Java-based.
2. contain patterns in each category. This is needed to compare the difference in smell-proneness among the different categories of design patterns.

To automate the data collection process and to make the process less error prone, we evaluated 11 popular design pattern detection tools [53, 65–74] as shown in Table 3. Unfortunately, these tools either detect few design patterns [65–70], have low precision [68, 71] or recall accuracy [66, 68] or do not specify precision and recall [65, 72, 74]. A similarity scoring-based tool, proposed by Tsantalis [53], covers 11 design patterns (less than 50% of GOF patterns) and has high precision and recall rates, however it does not differentiate between the instances of Adapter and Command patterns and between the instances of State and Strategy patterns. Our concern is that these pair patterns, i.e. Adapter/Command and State/Strategy are from different categories. Consequently, this might affect the results and conclusion of the study if any of them has significant relation with code smells. In addition, the tool extracts only the main participants of the patterns, i.e., it is not able to detect the role of each design pattern type. Hence, it is not suitable to be used in this research. Moreover, most of the surveyed tools, including this one, work under a condition that the system should be compiled successfully. Therefore, for the aforementioned reasons, and influenced by similar design pattern studies ([39], we used *some* systems from the P-Mart benchmark repository [60], as it satisfies our data requirements and is commonly used in design pattern research [75–77].

**Table 3:** Design patterns detection tools.

Tools	# of detectable design patterns	Precision%	Recall%
SPQR [65]	1	-	-
MARRPLE [66]	3	78.6	78.3
DP-Miner [67]	4	91–100	-
WOP [68]	4	57.3	54.5
DPRE [69]	6	62–97	-
DPJF [70]	8	100	80
Similarity Scoring [53]	11	100	95.9
DeMIMA[71]	13	34	100
Pinot [72]	17	-	-
PTIDEJ [73]	20	65	100
FUJABA [74]	All (GoF) patterns	-	-

P-Mart dataset is available as an XML-format file that consists of design patterns data for several Java-based systems; each system data is collected in a separate session by post-graduate students. To be aligned with the recent research, we collected the same five Java systems used by Elish and Mohammed [39] from the P-Mart repository. However, while parsing the XML file, we noticed that the repository contains more Java-systems (i.e., currently, P-Mart contains design patterns data for a total of 15 Java systems). Therefore, and to build a stronger empirical evidence, we doubled our systems to have five more systems. We randomly selected the new five systems. As a result, we collected seven main Java systems and three versions of DrJava system, as shown in Table 4. Table 4 provides the descriptive statistics for the used systems. The systems along with the analyzed data are available at our published dataset online (http://doi.org/10.5281/zenodo.3633081).

**Table 4:** Descriptive statistics on the analyzed systems.

Systems	Language	Release no.	# Classes	LOC
DrJava	Java	v20020619	215	47,617
		v20020703	238	52,870
		v20020804	267	61,844
JHotDraw	Java	v5.1	155	16,085
MapperXML	Java	v1.9.7	217	32,667
Nutch	Java	v0.4	165	37,106
PMD	Java	v1.8	446	52,302
Junit	Java	v3.7	78	6,517
QuickUML	Java	v2001	156	23,319
Lexi	Java	v0.1.1	24	10,005
Total # (All Systems)			1961	340,332

#### 3.4.2 Code smell detection

To detect the code smells for selected systems, we followed the approach of Walter and Alkhaeir [24] where they used inCode tool. However, in this paper, we decided to use “inFusion” tool (https://www.intooitus.com/products/infusion) [61] which is an extended version of inCode tool. inFusion can detect 22 code smells, 10 of which are identified by Fowler [11]. iPlasma [78] is an old version of it. Moreover, inFusion provides well-documented definitions of the detection rules and techniques used and their associated metrics with references. It supports visualization and refactoring.

In addition, due to high rate of false positives in most of detectors, we ran inCode tool [79] across data sample from the subject systems in order to validate detected instances. We use inCode tool as it has been applied in many studies of design pattern and code smells (e.g. [24]). In this sample, we considered smells to exist in a system if both tools could detect the same smell instance. This way, we make results of smell detection more reliable. We looked at 30% as random sample of smell instances (~65 instances) and found 4.1% (3 instances) as error rate. Most importantly, inFusion and inCode tools outperform other tools in terms of capability to detect smells regardless of compilation issues.

#### 3.4.3 Analysis of granularity at design patterns and code smells detection

Granularity analysis is the difficulty in tackling design patterns and code smells relations. Design patterns mainly refer to classes. On the other hand, code smells are related to several levels i.e. methods, classes, modules and packages. In this study, similar to [24], we analyze all instances of DPs and code smells at the class level. After mining smelly instances at method level over systems, we found only 14 instances (6.1%) of design pattern smelly methods, hence any analysis on method level will most probably lead to statistically insignificant results. Therefore, we assigned code smell instances that are at method level (i.e. Feature Envy, Refused Parent Bequest) to the classes they belong to. For example, the smelly instance of method: *com*.*lexi*.*lexiClass*.*lexiMethod()* was assigned to its class *com*.*lexi*.*lexiClass*. Smells that appear at other levels (e.g., packages and modules) were not considered in this study, as they do not have corresponding levels at design pattern side. For DPs, we only considered the main level of classes i.e. nested classes like inner/static classes were considered within the content of the parent classes since they have no relation to design patterns structure.

#### 3.4.4 Design patterns and code smell data

**Fig 2:**
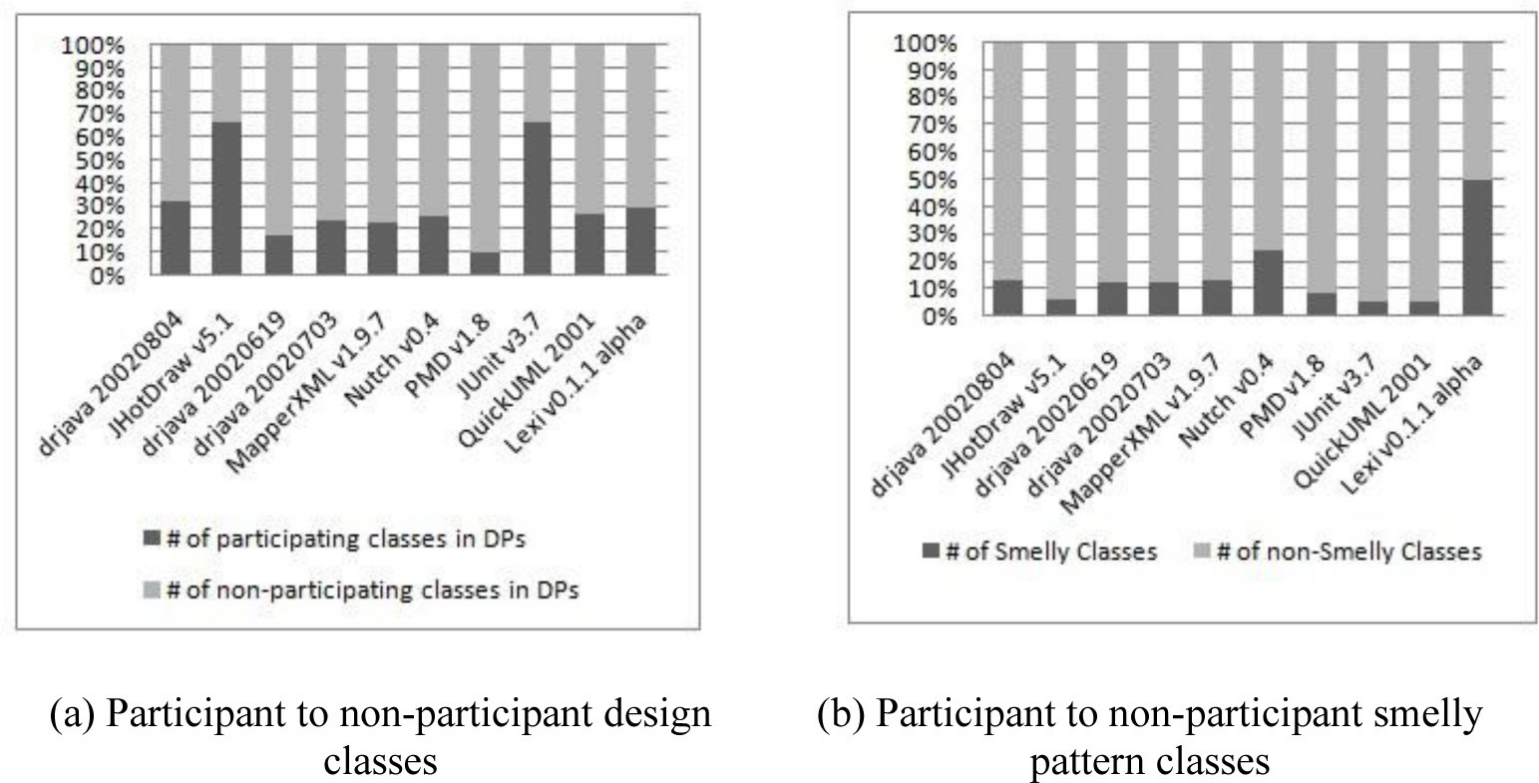
Statistics of participant to non-participant classes. (a) Participant to non-participant design classes. (b) Participant to non-participant smelly pattern classes.

Table 5 provides a statistical description for design patterns and code smell data while Table 6 reports the number of design pattern instances (motifs) in each of the subject systems. These tables describe both: (i) the number of participating classes in design patterns and (ii) the number of instances of each design pattern in each project, respectively. Design pattern instances range from 5 to 34 over the subject systems, as shown in Table 5, design pattern participant classes range approximately from 9.6% to 66.5%. The same thing is observed in relation to the number of smelly classes in the subject systems. The percentage of smelly classes ranges from 4.5% to 50% in the subject classes. Fig 2(A) and Fig 2(B) highlight these numbers. From Fig 2, we observe that while JHotDraw v5.1 has high number of DP classes (i.e. 103 DP classes, 66.5%), it has low number of smells (i.e. 8 classes, 5.5%). The same thing can be said for JUnit v3.7 project. However, other projects vary for the number of patterns and smells. For instance, PMD v1.8 project has low number of DP classes (i.e. 43 classes) while it has 35 smelly classes. Hence, we can superficially build a partial conclusion based on these figures. Next section explains the detailed methodology plan we follow to conduct the study.

**Table 5:** Statistics on smelly and design pattern classes.

Systems	# Classes	# & Percentage of DP Classes	# of Smelly DP Classes(SDP)	# of Smelly non-DP Classes(SnDP)	# &Percentage of Smelly Classes	# of Non-Smelly DP Classes(nSDP)	#of Non-Smelly non-DP Classes(nSnDP)	# & Percentage of Non-Smelly Classes
DrJava v20020804	267	85 (31.8%)	9	25	34 (12.7%)	76	157	233(87.3%)
JHotDraw v5.1	155	103 (66.5%)	5	3	8 (5.5%)	98	49	147(94.8%)
DrJava v20020619	215	41 (19.1%)	5	20	25 (11.6%)	36	154	190(88.4%)
DrJava v20020703	238	55 (23.1%)	5	23	28 (11.8%)	50	160	210(88.2%)
MapperXML v1.9.7	217	48 (22.1%)	6	21	27 (12.4%)	42	148	190(87.6%)
Nutch v0.4	165	41 (24.8%)	13	26	39 (23.6%)	28	98	126(76.4%)
PMD v1.8	446	43 (9.6%)	6	29	35 (7.8%)	37	374	411(92.2%)
JUnit v3.7	78	52 (67.7%)	2	2	4 (5.1%)	50	24	74(94.9%)
QuickUML 2001	156	41 (26.3%)	1	6	7 (4.5%)	40	109	149(95.5%)
Lexi v0.1.1 alpha	24	7 (29.2%)	2	10	12 (50%)	5	7	12(50%)
**Total # (All Systems)**	**1961**	**516(26.3%)**	**54**	**165**	**219(11.2%)**	**462**	**1280**	**1742(88.8%)**

**Table 6:** Design pattern instances in the subject systems.

Category	Patterns	drjava-20020804	JHotDraw v5.1	drjava-20020619	drjava-20020703	MapperXML v1.9.7	Nutch v0.4	PMD v1.8	JUnit v3.7	QuickUML	Lexi v0.1.1
**Creational Patterns**	Abs Factory					1				1	
	Builder				1			2		1	1
	Factory Method	1	3		1	1		3			
	Prototype		2								
	Singleton	8	2	8		3	1		2	1	2
**Structural patterns**	Adapter	2	1	2	1	2	2	1			
	Bridge	1					2				
	Composite		1			1		2	1	1	
	Decorator		1						1		
	Façade					1					
	Flyweight										
	Proxy	1		1	1			1			
**Behavioral patterns**	Chain of Responsibility										
	Command	2	1	2			2			1	
	Interpreter										
	Iterator	1		1			1	1	1		
	Mediator	1									
	Memento	1		1			2				
	Observer		2			1		2	3	1	2
	State	3	2		3						
	Strategy	3	4	1	1	1	2				
	Template	9	2		8	4	3	1			
	Visitor	1		1				1			
**SUM**	** **	**34**	**21**	**17**	**16**	**15**	**15**	**14**	**8**	**6**	**5**

## 3.5 Statistical tests

To achieve the research objectives, we used several statistical tests as summarized in Table 7. We used similar statistical tests used by the two most related studies to this study [24, 39]. The independent variables in this work are the classes participating in the design patterns and the dependent variables are the smelly classes. The details of the used statistical tests are given below. The main justification behind choosing these tests comes from the characteristics of these tests. In addition, the adopted tests in this study have been used in several previous related studies. The following descriptions of each test explains why they are suitable in this study. All p-values obtained in this study are at the 95% confidence level.

**Table 7:** Statistical tests used to achieve each objective.

Research Objective	Test Type	Test Objective
**Objective 1:** Empirical evaluation of code smell-proneness and smell frequency in design pattern classes versus non-design pattern classes at class level	Wilcoxon signed-rank [80]	Compare the significance of the overall data at class level
	Odd Ratio (OR) [81]	
**Objective 2:** Empirical evaluation of code smell-proneness in design pattern classes versus non-design pattern classes in design patterns categories (creational, structural and behavioral).	Kruskal Wallis [82]	Measure multiple groups (categories) of data
	Mann-Whitney U [84]	Compare the pairs of categories.
**Objective 3:** Empirical evaluation of code smell-proneness for the individual design motifs.	Kruskal Wallis test [82]	Compare the design patterns in each category
	Apriori algorithm [83]	Test the association rules analysis
	Manual source code-based analysis	To validate significant rules, if any and identify roles participating in the pattern

Wilcoxon signed-rank test [80] is a non-parametric test to compare the differences between two pairs of data.

We calculated the odds ratio (OR) [81] which specifies the probability of an event occurring. The odds ratio can be calculated based on two groups. The first group is the design pattern sample (p), while the other group is the non-design pattern sample (q). The ratio is given as follows:

$$OR=\frac{p/(1-p)}{q/(1-q)}$$ (1)

An odds ratio of 1 point to the equality of both samples. This indicates that the occurrence is equally probable in both samples, while the OR of points greater than 1 means that the first sample (design pattern sample) in the numerator is more likely to have smells. On the other hand, the OR value of points less than 1 indicates that the second sample more probably has smells.

The Kruskal-Wallis test [82] is utilized to compare multiple groups. In the context of this work, it is used to compare the differences among the design patterns of each category.

The Apriori algorithm [83] identifies the frequent item sets in the database and then it extends them to larger item sets that appear sufficiently in the database. The item sets identified by the Apriori algorithm can be used later to determine the association rules that have trends in the database, based on metrics such as Support.

Mann-Whitney U test is utilized to evaluate variations between different groups by making a comparison between two independent groups [84]. It is a non-parametric test that can facilitate a comparison between the classes that participate in the design patterns and the non-participating classes in terms of the disparity in smell-proneness.

## 4. Empirical study results

The objective of this section is to empirically evaluate the relationship between design patterns and code smells at different levels, i.e., class level, category level, motif level and role level.

### 4.1 Smell-proneness and smell frequency evaluation at class level

*RQ1*: *Are design pattern classes more smell prone than non-design pattern classes*? To answer RQ1, we evaluate the difference between participant and non-participant design pattern classes in terms of smell-proneness and smell frequency. This evaluation provides an insight on the impact of design patterns on the presence and frequency of smells.

#### 4.1.1 Smell-proneness evaluation

**Fig 3:**
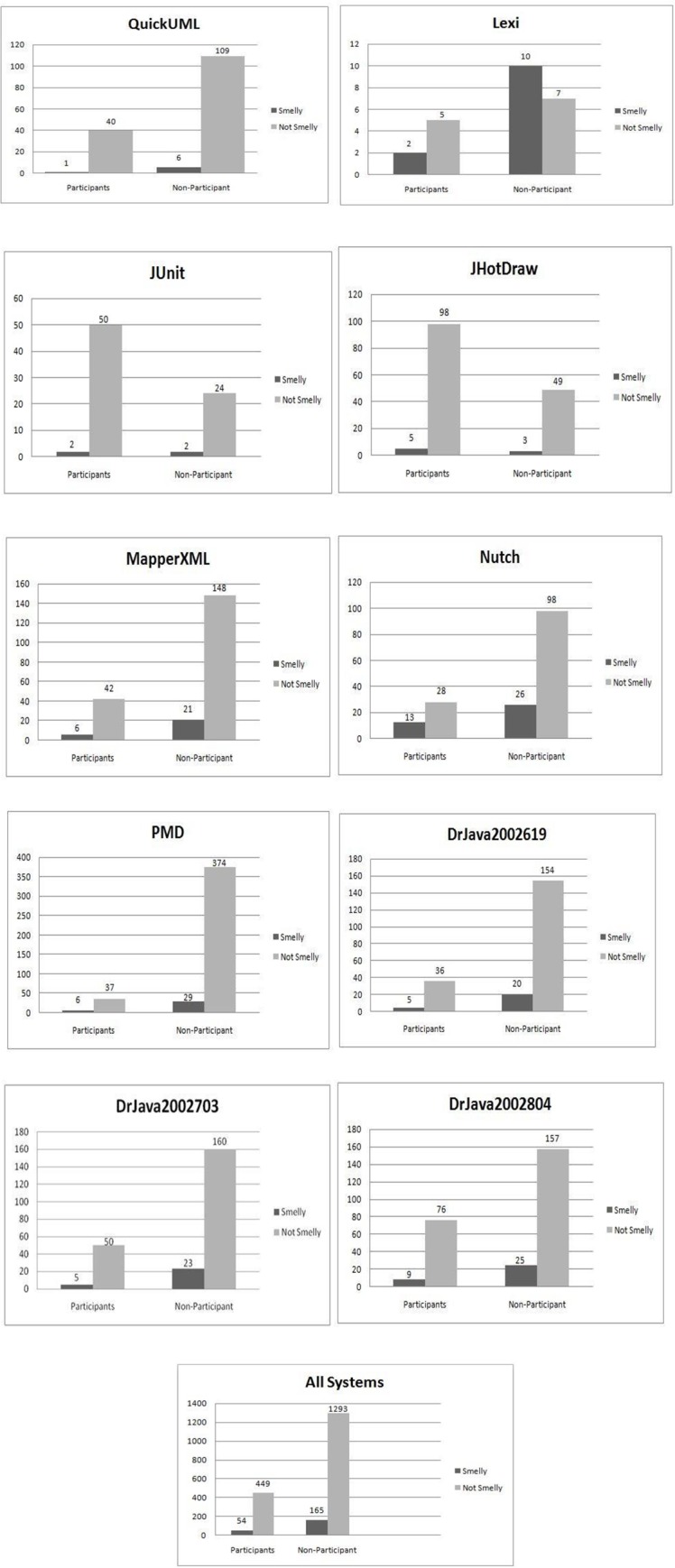
Smell-proneness comparison of participant vs. non-participant design pattern classes in all systems.

To achieve this goal, we compute the odd ration (OR) test. The same test has been used in other studies in the context of smells and changes [43]. OR test points to the possibility for an event to occur. Therefore, it could fit in this place as a start point for results. The evaluation results of smell-proneness in participating versus non-participating design pattern classes using the Odd Ratio test are shown in Table 8. It can be observed from that most of the subject systems show significant differences i.e. the smell events are more likely to be associated with the group of non-design pattern classes as the Odd Ratio (OR) has values less than one in most of the systems. Moreover, when all systems are combined, we found that the results still hold. However, only two values: the Nutch system and PMD system, have opposite results. The Nutch system covers 24.8% as design pattern classes while the PMD system covers only 9.6%. From Table 8, the OR value of the PMD system is more than 2. We note that as the PMD system has low instances of DP classes, it has a high value of OR. This can be interpreted as when there is a low number of design patterns, we might have a high number of smells. In addition, we can see from Fig 3 that the non-participating design pattern classes group is more likely to have a smell event than the participating design patterns group in most of the subject systems. In spite of having DP classes with less smell-prone than non-DP classes in the subject systems and to provide more clear confirmation, we conduct a more rigorous analysis of the data in the next sections.

**Table 8:** The odd ratio test analysis for smell-proneness evaluation of participant vs. non-participant design pattern classes groups.

Systems	Odd Ratio Value	Smell Event
QuickUML	.454	***<*1**
Lexi	.280	***<*1**
JUnit	.480	***<*1**
JHotDraw	.833	***<*1**
MapperXML	1.007	≈ 1
Nutch	1.750	*>*1
PMD	2.091	*>*1
DrJava2002619	1.069	≈ 1
DrJava2002703	.696	***<*1**
DrJava2002804	.744	***<*1**
All systems	.907	***<*1**

**Fig 4:**
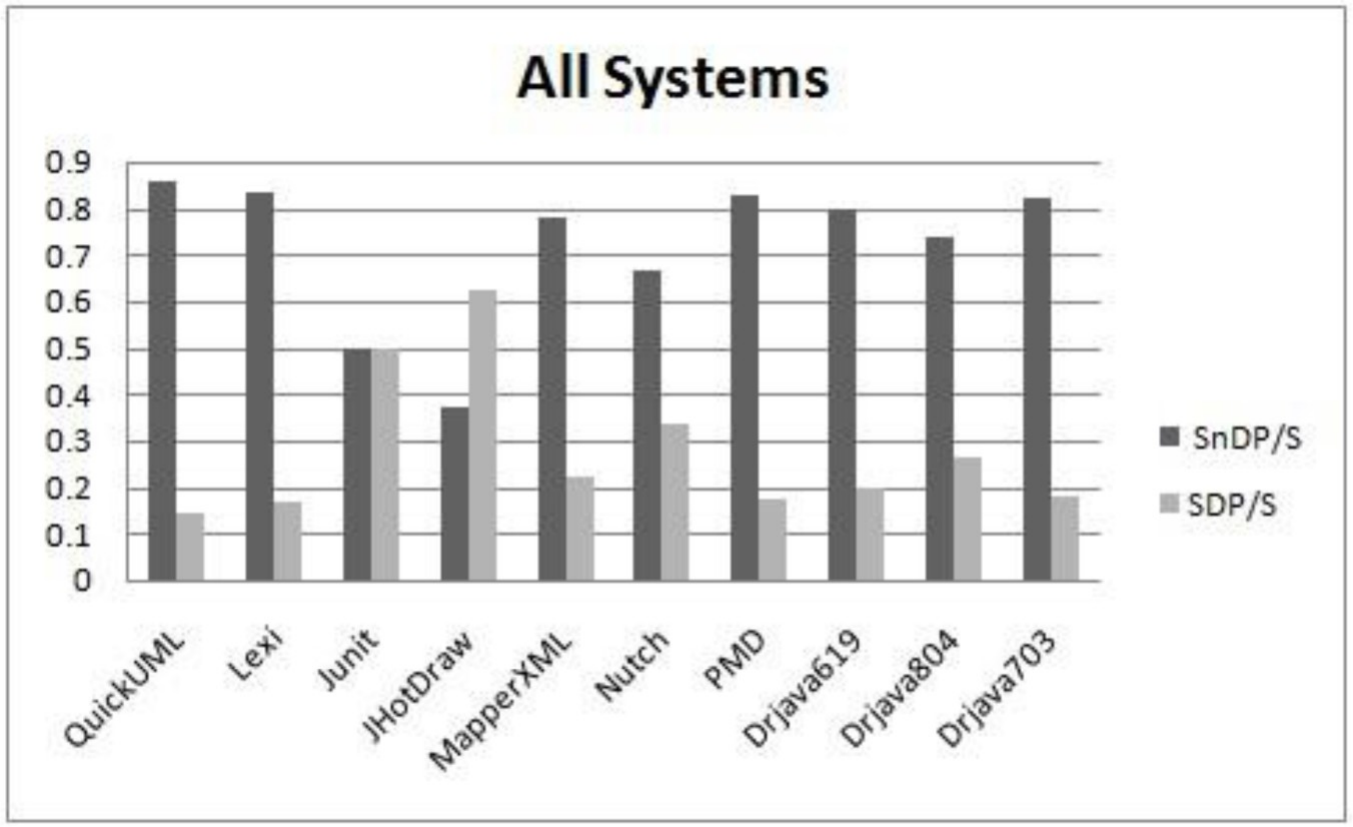
The respective values of smell-proneness in SDP and SnDP.

Given the non-normal distribution of SDP and SnDP samples, as shown in Table 9, for the classes of all the systems, we conducted more tests. From an inspection of the mean and median values, shown in Table 10, we can visually expect that the percentages of design patterns instances are smaller than non-design patterns instances in terms of smells, as presented in Fig 4. However, this expectation needs more verification. The Wilcoxon test is used as an effective and powerful alternative for the t-test which is used for normally distributed data. Due to the characteristics of the Wilcoxon test and the fact that combining smelly design pattern and smelly non-design pattern classes represents all smells, we associated each system with two values: SDP/S and SnDP/S, where S represents all smells in each subject system. The results are as follows: (z = -2.547, p-value = 0.011). Hence, the classes which participate in design patterns are less smell-prone than the classes not participating in design patterns for the subject systems, at a 95% confidence level.

**Table 9:** Test of normality.

Tested Variable	Group	Kolmogorov-Smirnov (Significance)	Shapiro-Wilk (Significance)
Smell-Proneness	Participant	***<*0.001**	***<*0.001**
	Non-Participant	***<*0.001**	***<*0.001**
Smell-Frequency	Participant	***<*0.001**	***<*0.001**
	Non-Participant	***<*0.001**	***<*0.001**

**Table 10:** Statistics of smell-proneness in all systems.

Metric	SDP/S	SnDP/S
Mean	.280	.719
Median	.211	.788
Std. Dev.	.161	.161
Variance	.026	.026

#### 4.1.2 Smell frequency evaluation

The Odd Ratio test cannot be computed on smell frequency data due to the nature of this test, as it requires data to be in a 2*2 table i.e. the data for the OR test should have four and only four cases. In the context of our data, we have two values: 1 or 0 for smell proneness data to identify whether a class is affected or not. While in the case of smell frequency data, we have a series of numbers e.g. 1, 4, 8, 12, 40 and so on. Thus, we cannot run the OR test over smell frequency data. Therefore, for smell frequency, we only use the Wilcoxon test.

**Fig 5:**
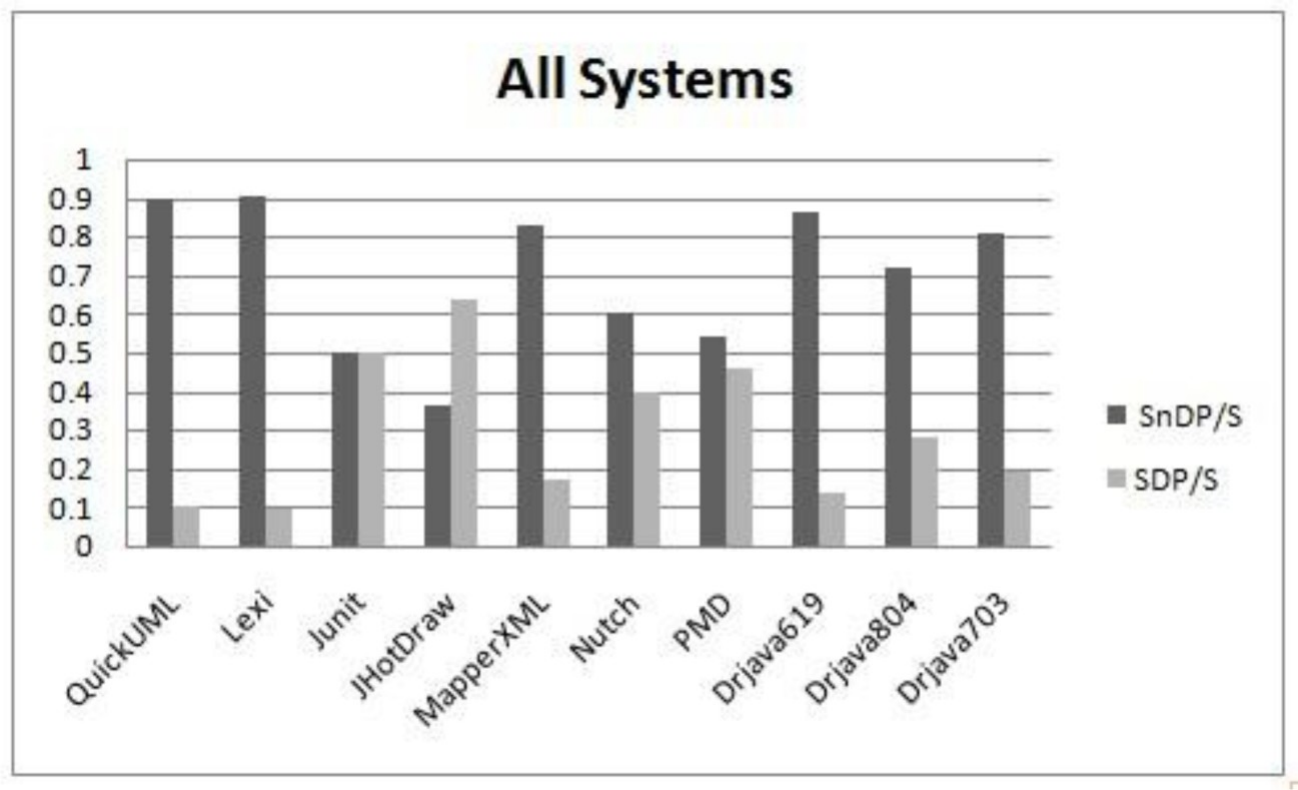
The respective values of smell frequency in SDP and SnDP.

To evaluate smell frequency using the Wilcoxon test, we followed the same procedure used in evaluating smell-proneness, given that SDP and SnDP are not normally distributed as shown in Table 9. Smell frequency indicates the number of smells associated per class in both groups: SDP and SnDP. The resulting values are shown in Fig 5. The respective mean and median values are shown in Table 11. The results are as follows: (z = -2.310, p-value = 0.021). Hence, the classes which participate in design patterns have less smell frequency than the classes which do not participate in the design patterns for the subject systems at a 95% confidence level. Interestingly, the results of smell-proneness and smell frequency are consistent. Therefore, in our next experiments, we apply our analysis based on smell-proneness data rather than smell frequency data, since most of the classes, i.e. ~ 85% in the subject systems have only one smell.

**Table 11:** Statistics on smell frequency in all systems.

Metric	SDP/S	SnDP/S
Mean	0.297	0.703
Median	0.238	0.762
Std. Dev.	0.190	0.190
Variance	0.036	0.036

### 4.2 Smell-proneness evaluation at the category level

To evaluate RQ2: Do code smells have significant differences when they present in the different categories of design pattern classes? We use the three design pattern categories as defined by the GoF: creational, structural and behavioral. To evaluate the differences in smell-proneness among these different categories, we identified 3 pairs to undergo statistical tests. Prior to making any comparison between the pairs, we conducted the Kruskal-Wallis test to find whether there is a difference among the categories. If a difference exists, we will go further and conduct the Mann-Whitney test to do comparison for each pair.

The pairs in our study are as follows:

1. Creational vs. Structural
2. Creational vs. Behavioral
3. Structural vs. Behavioral

In evaluating the differences in smell-proneness of classes that participate in the different design pattern categories, we did not consider the Nutch, JUnit and DrJavav2002703 systems since they are only associated with 1 class, 2 classes and 2 classes of the creational category, respectively. In addition, we ignored the Lexi system because the number of classes that participate in the structural design patterns is zero. Therefore, we ended up with 7 cases (systems) only including the all systems case.

**Fig 6:**
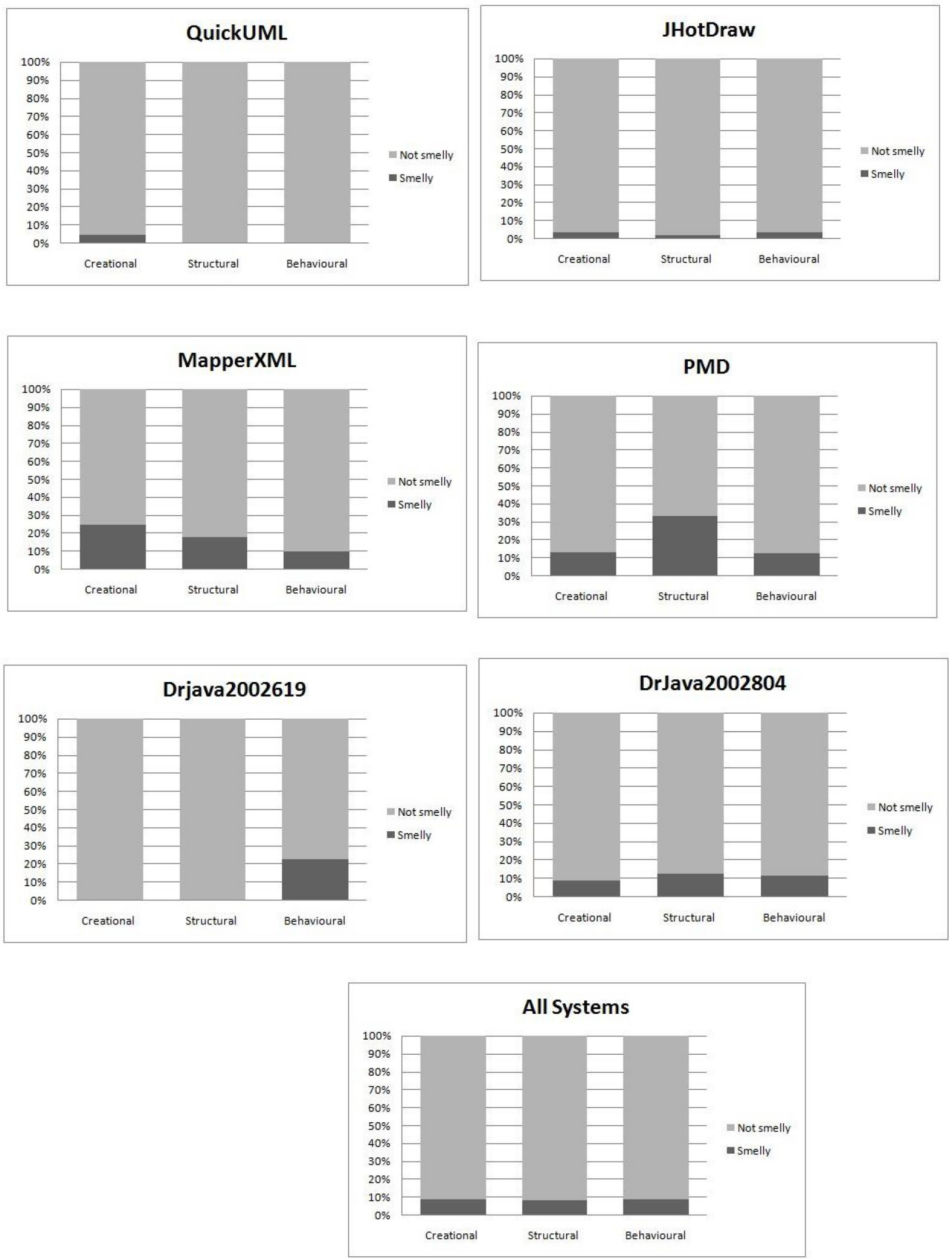
Smell-Proneness comparison of design pattern categories.

To evaluate smell-proneness at the category level, we used the Kruskal-Wallis test. As shown in Table 12 and from the visual inspection in Fig 6, the p-values obtained in this test have no significant values in all systems, even when all systems are combined at a 95% confidence level. Noticeably, the JHotDraw and DrJava2002804 systems have very close percentages of smelly design pattern classes in the categories with a maximum of 3.8% at the behavioral category and minimum of 2.2% at the structural category for the JHotDraw system and for the DrJava2002804, the percentage values of the categories: creational, structural and behavioral are 9.1%, 11.4% and 12.5%, respectively. The QuickUML and DrJava2002619 systems have no smells associated to the structural design pattern category. This observation might be due to the nature and structure of the structural category. According to [39], the structural category tends to be less change-prone. Moreover, based on the authors’ teaching experience in the field of object-oriented design patterns, students may have more understanding of the structural category compared to the other categories and thus it is easier to apply properly. The PMD and MapperXML systems have numerous variations of smells in the categories. Interestingly, when all the systems are combined, the categories, creational, structural and behavioral, have almost the same values of smelly design pattern classes at 8.8%, 8.5% and 8.9%, respectively.

**Table 12:** P-values of evaluation design pattern categories using the Kruskal-Wallis test.

Systems	p-value
QuickUML	0.417
JHotDraw	0.872
MapperXML	0.333
PMD	0.338
DrJava2002619	0.081
DrJava2002804	0.986
All Systems	0.987

Given the results on the differences in smell-proneness among the design patterns categories, we do not need to proceed with further test i.e. the Mann Whitney test. However, we conducted the Mann Whitney test for pairs of categories to confirm the results. The p-values obtained from the pairs are shown in Table 13. It is noted that the results have no significant p-values in all the pairs. This observation might indicate that design pattern categories result in the same level of smelly code. However, this makes a big claim that use of any type of design patterns may introduce similar results of smells because the software reliability is not associated only with smell-proneness, but several aspects could be involved. Therefore, this should be discussed in more fine-grained level. Consequently, there might be specific patterns in each category which have diverse impact from others. Some specific patterns might surpass other patterns in terms of their impact on producing smells. For example, in the structural category, the Facade pattern might have a strong connection to smells while the Composite and the Bridge patterns might have a weak connection. Hence, there is a need to test the impact of design patterns on code smells at the individual design motif level. Next section presents the impact of design patterns at the motif level.

**Table 13:** P-values of the evaluation design pattern categories using the Mann Whitney test.

System/Pair	Creational vs Structural	Creational vs Behavioral	Structural vs Behavioral
QuickUML	0.386	0.317	1.000
JHotDraw	0.657	0.967	0.612
MapperXML	0.552	0.139	0.372
PMD	0.193	0.961	0.221
DrJava2002619	1.000	0.146	0.078
DrJava2002804	0.871	0.894	0.929
All Systems	0.907	0.976	0.874

### 4.3 The impact of design patterns at the motif level

Design motif refers to several classes of different roles that participate in a design pattern. To evaluate RQ3, we test the impact of design patterns on code smells at the individual design motif level, as follows:

1. The difference in smell-proneness among the overall design patterns in each category using the Kruskal Wallis test.
2. If we find significant differences in the previous test, we evaluate the co-occurrence of each design pattern-code smell pair using association rules learning in the Apriori algorithm. The answer to this question might identify examples of the co-occurrence of DPs and code smells.

#### 4.3.1 Evaluating the differences in smell-proneness among design pattern categories

**Fig 7:**
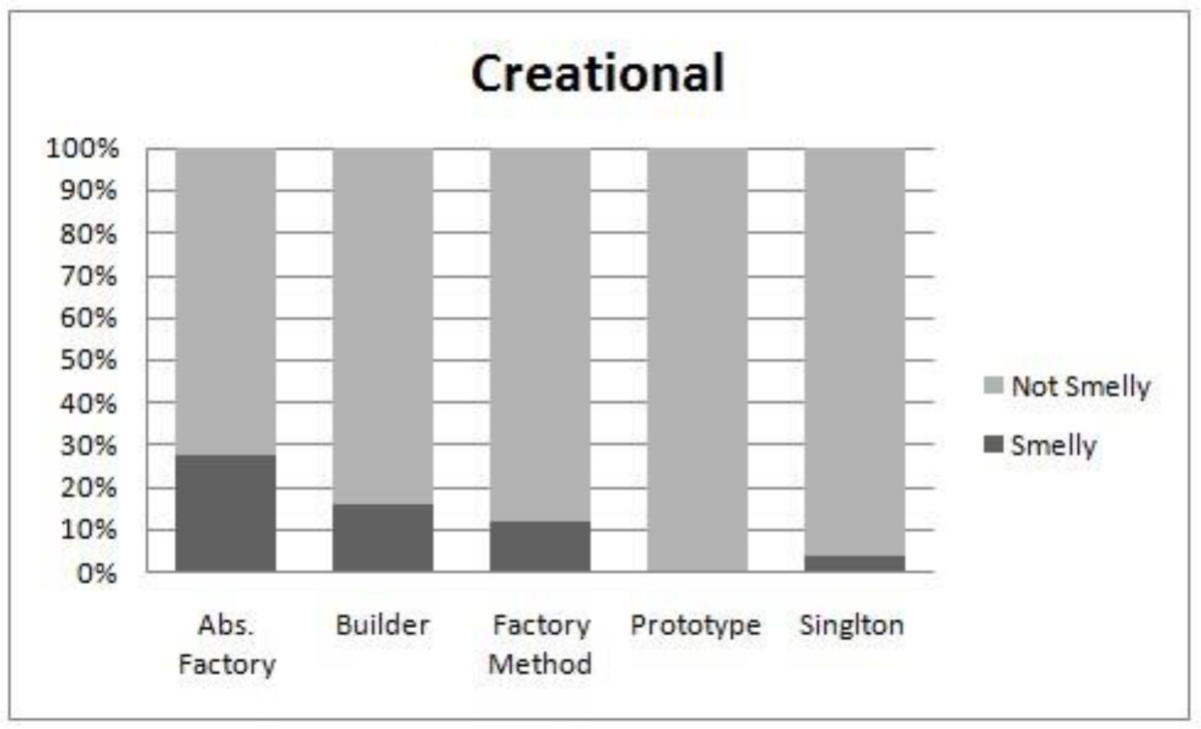
Comparison of smell-proneness in the creational category.

**Fig 8:**
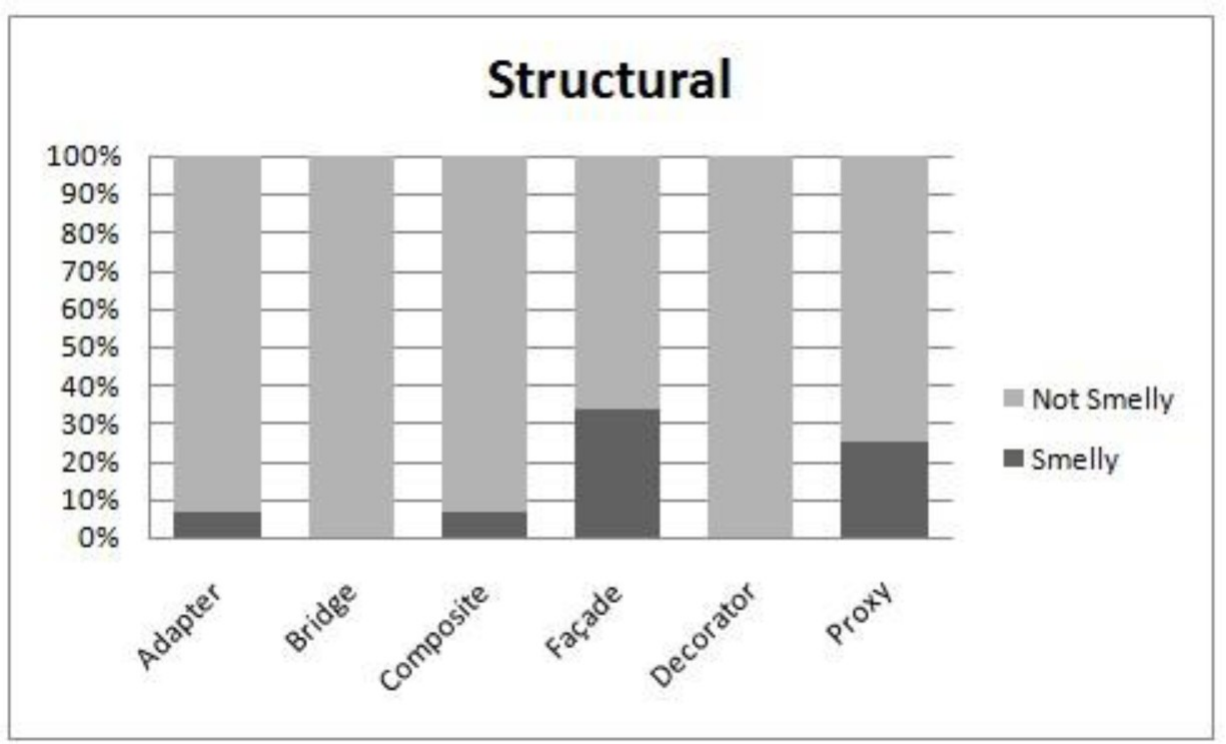
Comparison of smell-proneness in the structural category.

**Fig 9:**
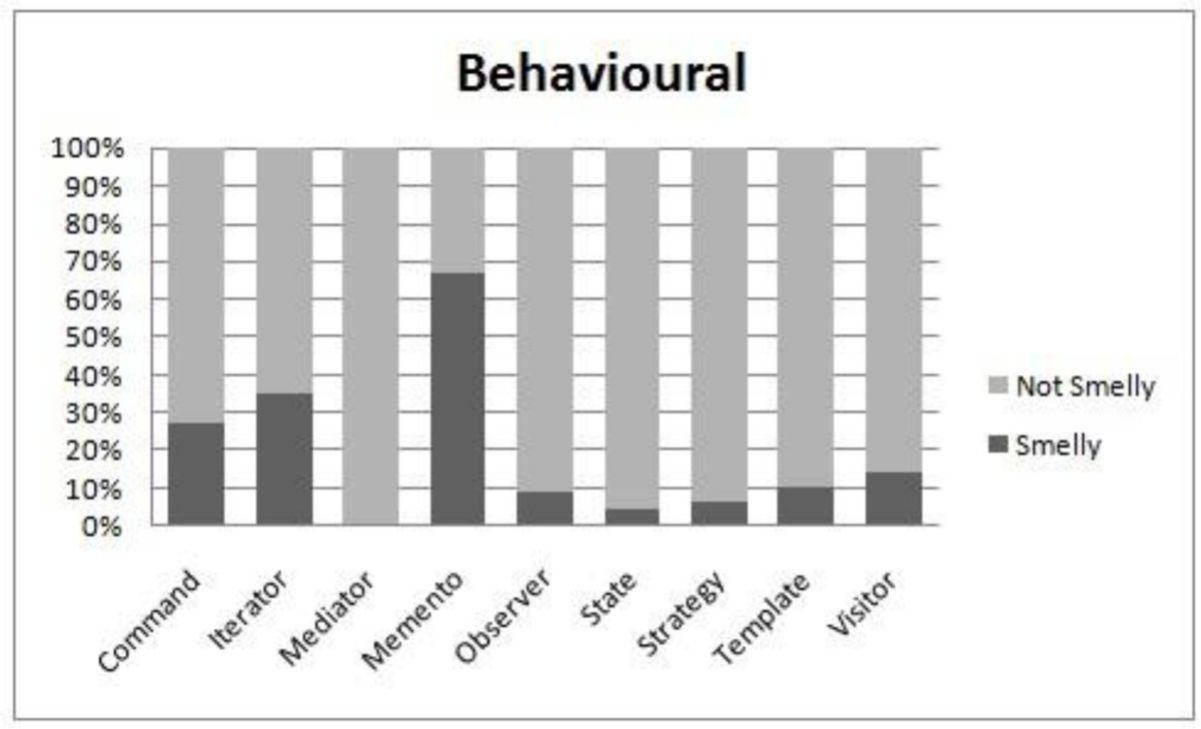
Comparison of smell-proneness in the behavioral category.

We perform the evaluation when all systems are combined. The reason for this is that the subject systems do not have the same set of patterns. A comparison of smell-proneness in each category i.e. creational, structural and behavioral, is presented in Fig 7, Fig 8 and Fig 9 respectively. Moreover, we used the Kruskal Wallis technique to test the significant differences among patterns in each category, as shown in Table 14.

**Table 14:** P-Value for the Kruskal Wallis test for each design pattern category.

Category of Patterns	p-value
Creational	0.048
Behavioral	**<0.0001**
Structural	0.007

We observe from Table 14 that each category has a significant p-value at a 95% confidence level which might indicate that the subject design patterns in each category have different behaviors in the context of their associations with smells. It is clearly observed from the obtained p-value in the behavioral category that some motifs have significant differences in terms of their associations to smells. However, some design patterns have more instances than others. For example, the Iterator and Memento patterns appear to be more smell prone than the Command pattern, as shown in Fig 9, although the Command pattern has 58 instances of patterns while the Iterator and Memento pattern has only 17 and 18 instances, respectively. In addition, the distribution of smells and design pattern instances is not equal. This observation might affect the conclusion of our results. Hence, further deep analysis is needed.

The next section provides a more rigorous analysis by applying data mining association learning rules to display possible significant relationships in the design pattern-code smell pairs. Association learning rules are vital concepts used to analyze the results [83, 85]. The rules are a mixture of attributes that occur in a dataset. In this study, the specific attributes in the environment we observed, consider two items which are design patterns and code smells. Three common measures were utilized in this place: Support [83], Confidence [83] and Conviction [85]. More details about the calculation of these measures is presented in the next section.

#### 4.3.2 Applying association rules for design patterns and code smell pairs

As discussed in Section 4.3.1, there is a need for further analysis to investigate the possible relationships that might associate specific design patterns with specific code smells. Consequently, the “Association Rules” concept is employed to identify such relationships. Table 15 presents the design patterns with their corresponding number of smells. The following acronyms are used for the column captions:

**Table 15:** Data of individual patterns with individual smells.

DP\CS	DC	DCL	RPB	SC	BL	IC	SD	ID	ED	GC	FE	TB	MC	SUM*	TOTAL^+^
Abs. Factory	2	-	-	1	1	-	-	-	-	-	-	1	-	5	18
Builder	-	1	1	-	-	-	2	-	-	-	1	-	-	5	25
Factory Method	2	1	-	3	4	1	-	1	2	1	1	1	-	17	95
Prototype	-	-	-	-	-	-	-	-	-	-	-	-	-	-	21
Singleton	-	-	-	-	1	-	-	-	-	-	-	-	-	1	27
Adapter	-	-	-	1	2	-	-	-	-	-	-	1	-	4	58
Bridge	-	-	-	-	-	-	-	-	-	-	-	-	-	-	28
Composite	2	-	1	2	-	1	-	1	3	-	1	-	-	11	102
Facade	1	-	-	-	-	-	-	-	-	-	-	-	-	1	3
Decorator	-	-	-	-	-	-	-	-	-	-	-	-	-	-	57
Proxy	-	-	-	1	-	1	-	1	2	-	1	-	-	6	8
Command	-	-	-	-	10	1	-	-	8	6	-	-	1	**26**	**58**
Iterator	-	-	-	-	3	1	2	-	-	2	-	-	-	8	17
Mediator	-	-	-	-	-	-	-	-	-	-	-	-	-	-	3
Memento	2	-	-	-	10	-	-	-	8	1	-	-	-	**21**	**18**
Observer	2	-	-	3	1	-	1	-	-	-	-	-	-	7	87
State	-	-	-	-	-	-	-	-	-	-	1	1	-	2	66
Strategy	-	-	-	1	-	1	2	-	-	-	1	-	-	5	90
Template Method	2	-	2	1	4	1	-	2	4	4	1	1	-	**22**	133
Visitor	-	-	-	-	-	-	-	-	-	1	-	-	-	1	7

* Smelly design pattern instances

+ All classes which participate in DP

DC-Data Class DCL-Data Clumps RPB-Refused Parent Bequest Class

SC-Schizophrenic Class BL-Blob IC-Intensive Coupling

SD-Sibling Duplication ID-Internal Duplication ED-External Duplication

GC-God Class FE-Feature Envy TB-Tradition Breaker

MC-Message Chains

The data presented in Table 15 can lead directly to certain observations:

- Only one smell was discovered to be contained in classes with the Singleton, Facade and the Visitor.
- Classes with the Prototype, Decorator and Bridge were discovered not to take place simultaneously with smells.
- The Blob, God Class and External Duplication smells are collocated with the Command patterns.
- The Blob and External Duplication are collocated with the Memento patterns.
- The Command and Memento patterns can take place simultaneously on a regular basis.
- Message Chain was identified in a single class which was taking part in the Command pattern. It was not observed in any other classes with other patterns.

A mixture of attributes in a data set can be expressed by association rules. The specific attributes are design patterns and code smells in the environment we observed. Two common measures were utilized to classify the dependency rules between the attributes which are *confidence* and *support* [83].

To compute these measures, the assumption was made that, in every system, an individual class represents a separate transaction. The following facts were subsequently established regarding the transaction: (i) it includes an occurrence of a smell and (ii) it includes an occurrence of a pattern. Every smell and every design pattern studied is referred to as an item set. Both metrics include values that range from (0–1), with higher values designating more important rules.

*Support* of an item set refers to the share of transactions which contains this item set, thereby demonstrating its significance [83]. For example, in the case where a system contains one hundred classes and ten of these classes exhibit the Feature Envy smell, this can be taken to signify that, regarding this system, the level of *support* is 10% for Feature Envy. As a further example, the level of *support* for the relationship between the God Class and the Factory Method illustrates the percentage of procedures which includes the God Class and the Factory Method. Consequently, the level of *support* is shown to be a gauge of the regular occurrence of an item in a relationship.

It is essential to be familiar with the generally agreed schemes for naming employed in the association rules: antecedent and consequent, so that the concept of *confidence* [83] can be properly understood. We regard design patterns to be the antecedent and smells to be the consequent. *Confidence* can be defined as the likelihood of observing the rules which result from the stipulation that the procedures include the antecedent. In other words, it represents the ratio between level of *support* for the association and the level of *support* for the antecedent. It is possible to determine the level of *confidence* using Eq 2. The value of *confidence* is usually greater if the result has a strong level of *support*. Due to this fact, there is a higher level of probability that there will be a correspondingly strong level of *support* for the association.

$$Conf(DP\to Smell)=\frac{Sup(DP\cup Smell)}{Sup(DP)}$$ (2)

In this part of the evaluation, we decided to test the rule which combines (1) antecedent: a design pattern on the left side and (2) consequent: a code smell on the right side, separately. To enable the weak rules to be documented for additional study, we set the minimal configuration values for *support* and *confidence* in Weka[86]. Several data mining techniques, such as the Apriori algorithm, are implemented using the WEKA tool[83]. The resulting association rules are equal to (20 * 13 = 260). Only 5 rules, shown in Table 16 were found to have low *confidence* (i.e. *<*95%). Most of the rules in our data set exhibit a weak relation between design patterns and code smells with high confidence level. However, results of other few rules also show that there is a positive association between the presence of specific design patterns and code smells, as shown in Table 16.

**Table 16:** List of significant association rules.

Rule no.	Rules
R1	*Command* ⇒*Blob*
R2	*Command* ⇒*GodClass*
R3	*Command* ⇒*ExternalDuplication*
R4	*Memento* ⇒*Blob*
R5	*Memento* ⇒*ExternalDuplication*

Another metric used for data mining purposes is called *Conviction*. It is an alternative to and more powerful than the *Confidence* metric [85] due to the way Conviction is calculated, as shown in Eq 3. The Conviction metric has the following characteristics:

- Support for both the antecedent and consequent.
- The value of the formula is 1 when the antecedent (DP) and consequent(smell) are completely independent.
- The value of the formula is infinite when the antecedent (DP) and the consequent(smell) are completely dependent.
- Values greater than 1 i.e. 1.01 indicate a possible association between the antecedent (DP) and the consequent (smell).

$$Conv(DP\to Smell)=\frac{Sup(DP)*(1-Sup(Smell))}{Sup(DP)-Sup(DP\cup Smell)}$$ (3)

We use *Conviction* to confirm the five rules observed in Table 16. Table 17 shows the conviction values for these rules. All the values are greater than 1.01, which confirms the previously defined rules in Table 16.

**Table 17:** The Conviction values of the identified rules.

Rule no.	Conviction Values
R1	1.16
R2	1.29
R3	1.11
R4	1.97
R5	1.61

#### 4.3.3 Source code-based validation technique at role level

In this section, we use source code-based analysis technique to validate the significant rules, and identify roles participating in the motif.

An explanation for our conclusion can be provided by the definitions and purposes of the Command and Memento patterns. Obviously, an excessive implementation of the pattern within the system evolution might potentially lead to the Blob and God class smells in Command and Memento patterns. To build our final conclusion and demonstrate causality between design patterns and code smells, we further improved our analysis by analyzing segments of source code that have co-occurrences of DPs and smells.

**Validation technique:** we consider Command pattern as an example. The Command pattern consists of several roles, receiver role class and some concrete command classes are among them. The receiver role class usually creates many methods to set and get the concrete command instances. Therefore, when the receiver is associated with many concrete commands, it leads to have many methods for that purpose. In addition, the receiver class has its own members that take care of other tasks. Therefore, the receiver class may become God Class. In particular, the Command pattern could relate to God class especially when it supports huge number of commands.

To provide a clear explanation of the characteristics and causes that should make the pattern more prone to this smell, we further analyze the data and the code of the subject systems. We selected DrJavav20020804 system since it has instances of God Class and Blob smells. We identified part of the class diagram of an instance of the Command pattern in the system using ObjectAid (http://www.objectaid.com) UML Explorer plug-in tool. We noticed among its classes that the *DefaultGlobalModel* class which plays the main role in the pattern is associated with high number of concrete command classes, (we identified at least 10 command classes). The class diagram is available at: http://doi.org/10.5281/zenodo.3633081. When we analyzed the class diagram, which is associated with the command part, we observed that *DefaultGlobalModel* class includes massive number of getter and setter methods related back to its concrete commands. Additionally, it contains other methods, which are in turn, have other responsibilities. Thus, the class *DefaultGlobalModel* becomes God Class. This was identified by inFusion tool as well. The most observed characteristic of the Command pattern class diagram in this system is that there are several categories of the concrete commands. For instance, some methods in concrete command classes take care of EventHandling issues, others are responsible for DocumentsHandler, Open/Save/Close Files, ClearHistory and so on. As these command classes are supported by and connected to *DefaultGlobalModel* class, therefore, this class has a high chance to become God Class in DrJavav20020804 system. Moreover, as it seems difficult to demonstrate such a relationship without running an experiment or observing the subsequent revisions in repository, we observed that the previous class diagram inhabits in the all three versions of DrJava system: v20020619, v20020703, v20020804.

Since most of the concrete command classes are concerned with different domains and purposes, we would suggest that a better practice in this case is to split these commands into several instances of the Command pattern. For example, commands related to Open Files should have their own instance. Hence, methods that currently belong to DefaultGlobalModel could be pulled out to a separate instance for Open Files responsibility. The same can be said about other types of command pattern. Table 18 summarizes the co-relationships between each test with respect to its result. A discussion and interpretation of the results are reported in the next section.

**Table 18:** Summary of results at design motif level.

Test Objective	Test Type	Test Results
Find the difference in smell-proneness among the overall design patterns in each category	Kruskal Wallis test	All categories have significant p-value at a 95% confidence level in the subject systems.
Explore the co-occurrence of each design pattern-code smell pairs	Learning association rules in the Apriori algorithm & source code-based analysis	Some rules show that there is a positive association between the presence of specific design patterns and code smells, as shown in Table 16. Moreover. We improved our analysis based on source code validation technique.

## 5. Discussion

In this section, we discuss the findings presented in Section 4. Also, we highlight the threats to the validity which may occur in this research.

### 5.1 Co-occurrence between design patterns and code smells

From Table 5, we can observe that the smelly classes are not widespread. It is estimated that only 4%-24% of classes in all systems are impacted by bad smells, with one exception which is the Lexi system (50%). The number of classes in the Lexi system is very low i.e. 24 classes only, 12 of which are smelly. Furthermore, 2.8% of all classes in the subject systems contain both design patterns and code smells.

**RQ1:** The relationship between smelly design pattern classes (SDP) and smelly non-design pattern classes (SnDP) for all systems, as shown in Fig 4 and Fig 5, is different across the selected systems. We confirm that (SDP ≤ SnDP) for most of the systems. However, for the JUnit and the JHotDraw systems, the situation is the opposite. An inspection of the data on the systems shows that only 2 classes in the JUnit system that had a schizophrenic smell were also represented in the Observer patterns. These relationships are not observed in other systems, which may imply that this is an isolated event due to suboptimal design choices. Eight classes in the Nutch system are affected by combination of Blob and External Duplication. For DrJava releases, DrJava-20020619, DrJava-20020703 and DrJava-20020804, we observe that as releases evolve in number of design pattern classes (41, 55 and 85 DP classes, respectively), the number of smelly classes also increases (25, 28 and 34 smelly classes, respectively). These results of DrJava systems might indicate that evolution of smelly pattern-systems could affect the following releases; patterns typically make certain changes easier and others harder. A pattern should only be applied when the flexibility it provides is required. Other systems like PMD project has low number of DP classes (i.e. 43 classes) while it has 35 smelly classes. However, PMD has high number of classes, i.e. 446 classes. Out of the 446 classes, 374 classes are not participating in any design pattern and code smells. This size of classes could clarify why PMD system has been affected by low number of smells. Therefore, the possibility of using size factor would provide more detailed information about the relation type between design patterns and bad smells. From Table 15 and a manual inspection of our systems (e.g., JHotDraw), we observe the following: Some smells are associated with some design patterns, e.g., State with Tradition Breaker, Factory Method with Data Clumps and Strategy with Schizophrenic. Other code smells are almost isolated from design patterns, e.g., Data Clumps and Message Chains.

**RQ2**: As shown in Table 12 and Table 13, the number of relevant cases varies from one pair to another. For instance, only 1 class as a creational category class is participating in the Nutch system, while the Lexi system does not have any structural design patterns. We observed that each category acts in the same way in terms of smell-proneness. This observation might tend towards the conclusion that the adoption of any of the design patterns might produce the same smelly software. However, the behavior category was found to be the highest category in terms of smell-proneness. Yet, specific patterns in each category have diverse impact on smells as compared to other patterns. For example, in the structural category, the Adapter pattern might have a strong connection to smells while the Composite pattern might have a weak connection, depending on their characteristics.

**RQ3:** We examined the most common smells and found the Blob, God Class and External Duplication to control the other smells in the systems. In the same way, the design patterns are not evenly distributed: The Template Method, Composite, Factory Method, Strategy, Observer and Command are the most employed instances in the subject systems. Such design patterns and code smell allotments comprise 61.9% of the total number of design pattern smelly classes. In our search for a solution to RQ3, we focused on identifying the possible strong associations between each design pattern and smell in the data set, characterized by “Association Rules”. Within a rule, we have a design pattern as an antecedent on the left-hand side, while a code smell plays the role of a consequent. Clearly, most of the important rules we discovered display the mutual exclusivity of design patterns and code smells. However, some rules represent a strong association between individual patterns with certain code smells, as shown in Table 16. It can be seen from Table 15 that Singleton, State, Strategy, Adapter and Decorator are patterns which are not allotted with smells generally, while this is the opposite for the Command and Memento patterns. As illustrated in Table 16, Command patterns are associated to the Blob, External Duplication and God Class smells, whereas the Memento patterns are related to Blob and External Duplication, with the God Class being the exception. Table 17 shows the conviction values for these rules. Our manual investigation observed that Command and Memento co-exist in the classes. Additionally, Blob smells co-occur with External Duplication smells in the subject systems. To further support our conclusion, we added a validation technique in Section 4.3.3. The technique is source code-based analysis technique and it confirms the results of co-occurrence between specific DPs and smells in some cases. Table 19 shows the proposed RQs with their answers.

**Table 19:** Summary of RQs and their answers.

Research Question	Question Answer
RQ1: Are design pattern classes more smell-prone and more frequent than non-design pattern classes?	Classes participating in design patterns have less smell-proneness and smell-frequency than classes not participating in design patterns in the subject systems.
RQ2: Do code smells have significant differences in terms of proneness when they are present in the different categories of design pattern classes?	Every design pattern category act in the same way in terms of smell-proneness in the subject systems. Yet, some patterns appear to be more smell prone than others. Therefore, we propose RQ3.
RQ3: Are the participant classes in a specific individual design motif more smell prone for a specific smell?	A weak relation between the presence of most design patterns and the absence of most code smells. However, we observed that some patterns are associated with certain smells. The most noteworthy cases are the Command pattern with Blob and God Class smells. In addition, the Memento pattern was discovered to be connected to Blob and External Duplication smells.

### 5.2 Practical implication

In the following subsection, we highlight the most important implications for developers, researchers, and tool builders driven from our findings.

#### 5.2.1 Developers

The co-occurrence information of design patterns and code smells help developers prioritize their code reviews. Depending on the relationship between design patterns and code smells, developers can give different priorities to different parts in the code. For example, if there is a positive relationship between design patterns and code smells, developers need to give prioritization to reviewing code fragments that contain design pattern classes. On the other hand, if the relationship is negative, develops should give the prioritization to non-design pattern code fragments. Therefore, the numbers of the patterns-based classes could serve as an indicator for code smell occurrences. Such numbers may correspond to proportions of code smells which in turn could be provided higher or lower priority based on the findings in studies, e.g., Command patterns classes could be given major priority for code reviewing since our results indicate a potential relationship between Command design patterns and code smells such as, Blob, External Duplication and God Class smells. Other unrelated patterns-smells association could gain lower priority. The parts with lower priority do not lead to ignoring them completely; however, code-review efforts by developers could focus more on modules other than pattern-classes.

#### 5.2.2 Researchers

In our study, we built statistical models relating design patterns to a dependent variable representing the presence of code smells. However, factors other than design patterns could also explain the presence or absence of code smells. For example, the size factor can explain the presence or absence of large and/or complex classes, e.g., Blob smells. Also, coupling factor can explain the presence or absence of Feature Envy or Message Chains smells. Therefore, researchers should explore such factors that may explain the extent to which classes participating to design patterns are smell-prone. To this aim, researchers should consider such control factors in the statistical models, as a consequence, it will help clarify the extent to which the presence of a design pattern contributes to the smelliness of code components less/more than other factors or whether design patterns are simply a co-occurrent phenomenon behind the presence and absence of code smells.

#### 5.2.3 Tool builders

Using information related to the relations between patterns and smells can be useful to identify the availability of smells somewhere in a software program. For example, knowing that most design pattern classes are not associated to code smells can concentrate on locating smells in specific components of the code. On the other hand, including patterns data into smell detection tools can also help on the other way around. For example, Aversano et al. [34] stated that design pattern classes that have functional role in systems are subject to intensive changes. Hence, co-occurrence of design patterns and code smells could affect these classes to be even more change and defect prone. Consequently, smell detection tools should provide more attention to such classes that could be worthy of more maintenance efforts.

### 5.3Threats to validity

**Construct validity** focuses on the ability to measure what we claim to analyze. The process of design pattern and code smell detection is regarded as being of special importance in this study. The various detection tools for design patterns and code smells can produce both false positive and false negative cases. However, we use *some* systems from the P-Mart repository to reduce this threat as it was produced using different validation phases such as: studies in the literature [36]; Ptidej (pattern trace recognition, discovery, and development in Java) tool used to classify design patterns [73]; and validation projects for undergraduate and graduate students. Therefore, the potential occurrences of both false negative and positive cases of design patterns in the P-Mart repository are reduced by employing these different sources. Also, due to high rate of false positives in most of smell detectors (as mentioned in section 3.4.2), in addition to using inFusion tool, we ran inCode tool [79] across data sample from the subject systems in order to validate the detected instances. We used inCode tool as it has been applied in many studies of design pattern and code smells (e.g. [24]). This way, we make results of smell detection more reliable and mitigate the false positive and negative cases.

Apart from the statistical models used in this study for evaluation, the possibility of using technical analysis and procedures such as correlation and regressions would fit to have more detailed information about the relationship between design patterns and bad smells. Given that the number of smelly DP classes in our dataset is very low (median is 5) and the percentage of the smelly classes is also not high (median 11%)), applying linear regression or multilinear regression is not suitable for a significant correlation or regressions.

Another possible threat comes from the adopted granularity analysis of methods matching DPs and smells at class level. Considering further complex model of granularity analysis might influence the results. Another threat is related the data analysis procedure adopted in this study. For instance, and due to the non-normal distribution of the variables, i.e. SDP and SnDP, we used non-parametric tests as replacement of the t-test. Consequently, we could not determine the power of applying t-test. Considering the density of smells in a system might also impact our results and conclusion. Hence, it is another control factor that needs to be considered. However, to mitigate, we considered smell frequency evaluation at class model. The results of smell frequency and smell proneness were found to be consistent. Moreover, the majority instances of smell in our dataset (almost 85%) have only one smell.

**Internal validity** can be defined as the ability to draw conclusions from the relationship between code smells and design patterns. Our study focuses on determining the potential association between design patterns and code smells, i.e., most design pattern classes are likely to have less presence of code smells. However, it is possible that other factors might impact the presence or absence of code smells. Our study does not focus on studying such factors; our scope in this work is limited to determine if there is an association between design patterns and code smells. Future work can investigation the factors that may impact the presence and absence of code smells.

**External validity**: the external validity of this study is endangered by the characteristics of the subject systems. Every subject system is open-source and has been created solely using the Java programming language. Thus, it is necessary to further explore the design patterns together with commercial systems developed in different programming languages and different software domains. To this end, this study should be regarded as an initial step which will help in promoting additional replications. The data set used in this research is available online to provide insightful information of various results on design patterns and code smells. This could be helpful for other researchers to investigate more about quality attributes of software engineering. Another external threat to validity is the issue of using small size dataset. We acknowledge that having large-size systems would give more confidence to our results. However, the reason for choosing these systems is the availability of their design patterns data in the P-Mart repository that is a manually validated dataset.

**Conclusion validity** can be defined as the extent to which the conclusions are made at the design level and the category level in ten different instances (i.e., ten subject systems in addition to the situation which occurs if every system is united). Most of the subject systems have a relatively small number of smelly classes (i.e., the number of smelly DP classes is very low (median is 5) and the percentage of the smelly classes is also not high (median 11%)). Conversely, two of the cases (Lexi and JUnit) have a comparatively small number of classes and examples of design patterns. Therefore, different conclusions may be arrived at by taking into consideration a larger number of cases and systems derived from different criteria. Concerning detection process using tools and data, we emphasize that the collected instances of DP and smells were only used to control statistical analysis and association rule-based learning as a starting point and not to state a final conclusion. However, our final conclusion was based on the source code analysis of the parts affected by co-occurrences between DPs and smells. Future work may consider validating bigger systems (e.g., Eclipse) to provide researchers the ability of conclusion generalizability.

## 6. Conclusion and future work

We explored the co-existence of design patterns and code bad smells. We started the study by collecting design patterns and code smell data. For the design patterns, the P-Mart repository was used. For the code smell detection, we used the inFusion tool over the ten open-source projects available in P-Mart.

The analysis of the study was conducted on three levels: class, category and individual pattern levels. At the class level, we found that classes participating in design patterns display less smells than classes not participating in design patterns. Smell-proneness and smell frequency were both considered at the class level, both showing consistent results.

At the category level, the results show that there are almost no significant differences between categories in terms of smell-proneness in the subject systems. However, at the level of individual design motifs, specific examples of design patterns’ connections with smells were discovered using association rules metrics, support and confidence. Although most of the rules showed a weak relation between the presence of design patterns and the absence of code smells, it was observed that there is a connection that could potentially facilitate the production of bad smells. The results show that the most noteworthy cases are the Command pattern with Blob and God Class smells. In addition, the Memento pattern was discovered to be connected to Blob and External Duplication smells. On the other hand, we observed that the Decorator patterns were significantly not connected with smells.

The results in this study contradict the perception that design patterns and code smells are mutually disconnected. The study presented a positive relationship between them. The observed results add to practitioners’ knowledge new causes and effects of code smells that could impact their presence.

Future studies can target reproducing an empirical study in the context of enterprise development, which could lead to additional data and consequently more statistical significance. In addition to altering the target systems, there is potential to employ additional detection tools that could produce superior results and may also lead to the detection of additional or possibly different instances of code smells. To ensure the success of this method, it is recommended that it be conducted in a completely controlled environment. This requires that the systems are familiar to and properly understood by the researchers. Under these conditions, the documentation of the design patterns employed must take place. In addition, another requirement could be that the system is not too big, which might also facilitate more accurate manual identification of bad smells. Moreover, future work can consider bigger systems other than those in the P-Mart repository, such as Eclipse, which makes the use of correlation and linear or multiple regressions analysis possible. This analysis would provide more detailed information about the relationship between design patterns and bad smells.
